# Preparation, solubility, and anti-inflammatory effects of a complex of diphenylcyclopropenone/β-cyclodextrin derivatives as the treatment of alopecia areata

**DOI:** 10.3389/jpps.2024.13230

**Published:** 2024-08-13

**Authors:** Yutaka Inoue, Kaede Yoshino, Suzu Kudo, Nao Kodama, Hajime Moteki, Mitsutoshi Kimura

**Affiliations:** ^1^ Laboratory of Nutri-Pharmacotherapeutics Management, Faculty of Pharmacy and Pharmaceutical Sciences, Josai University, Sakado, Japan; ^2^ Laboratory of Clinical Pharmacology, Faculty of Pharmacy and Pharmaceutical Sciences, Josai University, Sakado, Japan

**Keywords:** diphenylcyclopropenone, ground mixture, inclusion, solid-state, β-cyclodextrin

## Abstract

**Purpose:**

To investigate the preparation of inclusion complexes of diphenylcyclopropenone (DPCP)/β-cyclodextrin (β-CD) derivatives using a three-dimensional (3D) ball mill, and verify the inclusion behavior of the solid dispersion. Additionally, we aimed to investigate the effect of DPCP/β-CDs complex formation on the spleens of male C57BL/6 mice in terms of anti-inflammatory effects.

**Methods:**

The inclusion complexes of DPCP with β-CD and hydroxypropyl-β-cyclodextrin (HPβCD) were prepared using a 3D ball mill. Powder X-ray diffraction (PXRD) and Fourier-transform infrared spectroscopy (FT-IR) were used to evaluate the solid-state properties. The solubility of the prepared DPCP/β-CD and HPβCD complexes and the intermolecular interaction between DPCP and β-CD derivatives in solution were assessed using 1H nuclear magnetic resonance (NMR). Furthermore, the anti-inflammatory effects of DPCPs in the prepared DPCP/CD complexes were investigated using spleens from male C57BL/6 mice, with measurement of interferon gamma (IFN-γ) secretion as an endpoint. Additionally, the protective effects of each drug on NIH-3T3 cells exposed to ultraviolet (UV) irradiation were examined.

**Results:**

Solid-state characterization confirmed the formation of inclusion complexes in the 3D ground mixture (3DGM) (DPCP/β-CD = 1/1) and 3DGM (DPCP/HPβCD = 1/1) complexes through PXRD and IR analysis. The solubility of 3DGM (DPCP/β-CD = 1/1) and 3DGM (DPCP/HPβCD = 1/1) was 17.5 μg/mL and 58.4 μg/mL, respectively, indicating higher solubility than that of DPCP alone. NMR analysis of 3DGM samples suggested that DPCP/β-CD and DPCP/HPβCD form inclusion complexes at a molar ratio of 1/1 but with different inclusion modes. Regarding the anti-inflammatory activity of DPCP, 3DGM (DPCP/HPβ-CD) showed anti-inflammatory effects at lower doses compared to 3DGM (DPCP/β-CD) in terms of IFN-γ and NIH-3T3 cells injured by UV irradiation.

**Conclusion:**

We successfully formed inclusion complexes of DPCP/β-CD and DPCP/HPβCD using the 3D ground mixture method. NMR analysis suggested that DPCP/β-CD and DPCP/HPβCD form inclusion complexes at a molar ratio of 1/1 but with different inclusion modes. The anti-inflammatory activity of DPCP was more pronounced in 3DGM (DPCP/HPβCD) at lower doses compared to that in 3DGM (DPCP/β-CD), indicating that the HPβCD derivatives were more effective in enhancing the anti-inflammatory properties of DPCP.

## Introduction

Alopecia areata (AA) is an inflammatory disease with a genetic and immunological basis [1]. It is an autoimmune condition characterized by the expansion of circular hair follicle tissue and the formation of alopecia patches that experience recurring exacerbation and alleviation. Symptoms of AA include the presence of a dense infiltrate of primarily lymphocytes in the perifollicular area. Additionally, there is increased production of interferon gamma (IFN-γ) and interleukin-15 by lymphocytes in the epilation lesions. Furthermore, activation of CD8-positive NKG2D-positive cytotoxic T cells triggers an autoimmunological response targeting autoantigens derived from hair follicles [2, 3].

AA is the most common form of hair loss disorder and significantly impacts the appearance of hair, leading to concerns for patients and a notable decrease in their quality of life. While local steroid injection therapy is the primary treatment for AA, cases with an inadequate response are recommended to undergo local immunotherapy with diphenylcyclopropenone (DPCP) [4].

A study investigating the effectiveness and safety of DPCP, an immunomodulatory treatment, demonstrated a significant correlation between the severity of alopecia and treatment outcomes [5]. However, DPCP presents challenges due to its insolubility in water (solubility: 18.9 μg/mL) and issues related to its preparation and storage methods. Currently, DPCP is stored in light-shielded bottles in clinical settings and dissolved using acetone. Acetone causes skin irritation and is highly volatile, leading to potential changes in DPCP concentration and difficulties in storing diluted solutions [6]. Additionally, there are concerns regarding the exposure of DPCP to individuals involved in the drug preparation process.

β-cyclodextrin (β-CD) is a naturally occurring cyclic oligosaccharide composed of seven α-D-glucopyranose units linked by α(1→4) bonds. It possesses a conical structure with an outer hydrophilic and an inner hydrophobic region. To enhance its inclusion ability and physicochemical properties, several chemically modified cyclodextrins have been developed. One commonly used non-toxic cyclic oligosaccharide carrier is HPβCD, which is listed as a drug ingredient by the Food and Drug Administration [7]. HPβCD reportedly improves the stability and bioavailability of guest compounds, as well as reduces volatility by providing protection against degradation [8–11].

β-CD can form molecular complexes with various compounds, including prostaglandin E (PGE) [12]. Studies have also demonstrated that paclitaxel complexed with CD exhibits high water solubility and remarkable stability [13]. In the case of hydroxypropyl-β-cyclodextrin (HPβCD), the formation of an inclusion complex with isoflavone compounds reportedly enhances skin permeability [14]. Consequently, there are numerous examples of pharmaceutical applications utilizing β-CD derivatives [15, 16].

Various methods have been reported for the preparation of inclusion complexes, including the coprecipitation method [17], kneading method [18], freeze-drying method [19], and mixing and milling method [20]. One notable complex preparation method is the 3D mixing and milling method, which utilizes a 3D ball mill, a novel milling technique that has gained attention in recent years. The 3D ball mill features two rotary axes, one vertical and one horizontal, and utilizes the entire inner surface of a spherical container. This design reduces frictional heat generation and enables highly uniform milling and mixing in a short time [21].

In our previous work, we reported improved complex formation, solubility, and antioxidant capacity by using the 3D ball-milling technique to prepare mixtures of CDs with aglyconic isoflavones, specifically daidzein and genistein in the context of soy isoflavones [22]. Based on these findings, we propose that the preparation of inclusion complexes of DPCP with CDs using this innovative ground mixture technique could enhance the solubility of DPCP in the treatment of AA. This approach would allow for the preparation of DPCP at the time of use without the need for organic solvents, such as acetone.

Furthermore, the development of new formulations that consider factors, such as patient skin irritation during treatment, could serve as a foundation for future clinical applications. This method could potentially improve patient comfort and compliance, reduce the risk of adverse reactions, and streamline the preparation and storage of DPCP solutions.

In this study, our objective was to enhance the solubility and anti-inflammatory effects of DPCP, focusing on its inclusion complexes with β-CD and HPβCD using the 3D ball milling method. The solid-state properties of the complexes were evaluated using techniques such as powder X-ray diffraction (PXRD), Fourier-transform infrared spectroscopy (FT-IR), and scanning electron microscope (SEM). Additionally, the solubility and intermolecular interactions were assessed using ^1^H nuclear magnetic resonance (NMR) to gain insights into the solubility and interactions between the components.

The anti-inflammatory effects of DPCP in the prepared DPCP/CD complexes were investigated by culturing the spleens of male C57BL/6 mice and measuring IFN-γ levels in the culture medium. Furthermore, the protective effects of each drug on NIH-3T3 cells, which were injured by ultraviolet (UV) irradiation, were examined.

## Materials and methods

### Materials

**FIGURE 1:**
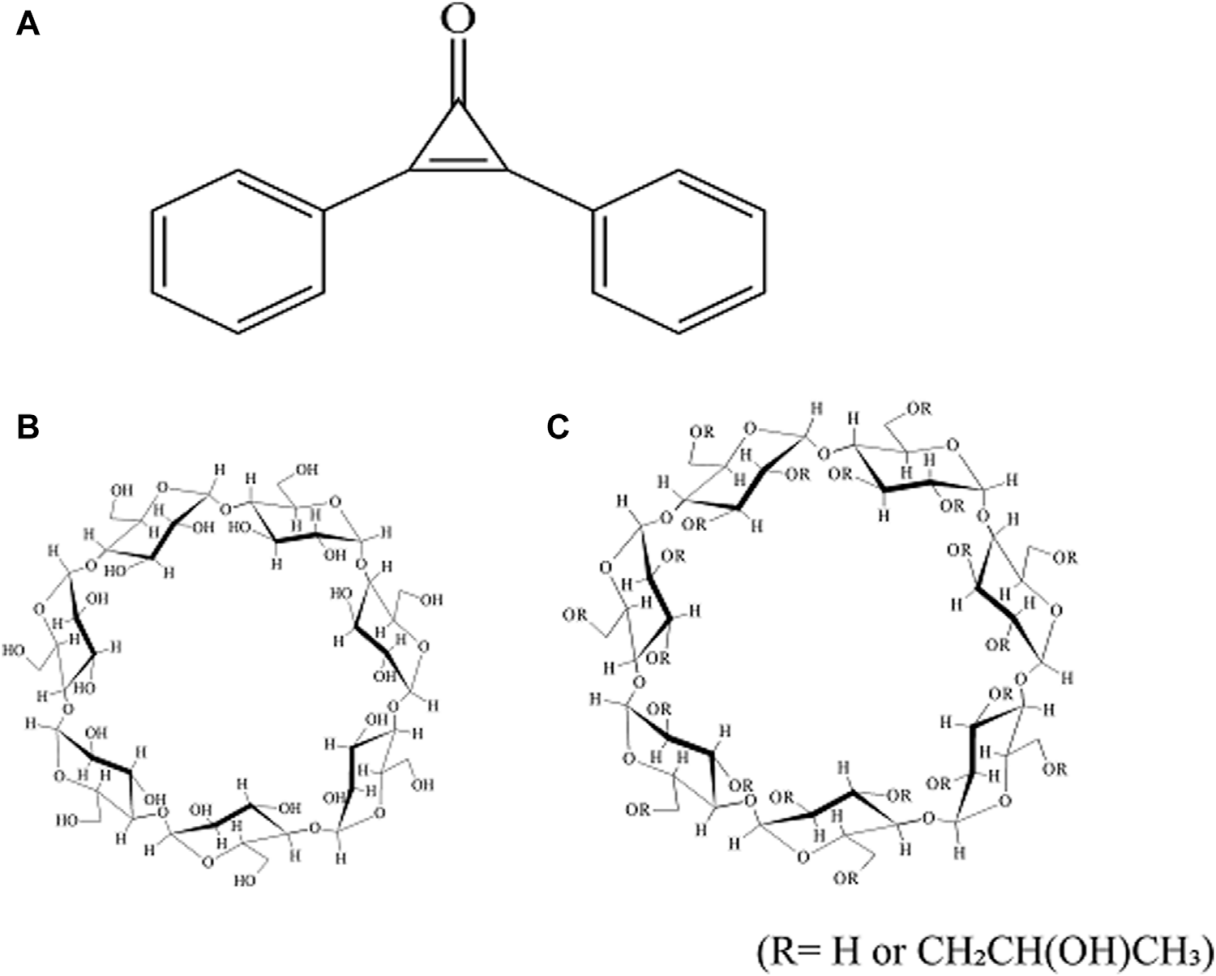
Chemical structure of DPCP, β‐ CD, and HP β CD. **(A)** DPCP, **(B)** β CD, **(C)** HP β CD. DPCP, Diphenylcyclopropenone; HPβCD, hydroxypropyl-β-cyclodextrin; β‐ CD,β-cyclodextrin.

DPCP (Lot No. LEH3622) was purchased from Fujifilm Wako Pure Chemical Corporation (Tokyo, Japan) (Figure 1). β-CD was supplied by Cyclo Chem Co., Ltd. (Kobe, Japan) and stored at 40°C with 82% relative humidity for 7 days. All other reagents used, including those for NMR analysis, were obtained from Fujifilm Wako Pure Chemical Corporation.

### Sample preparation of physical mixture and mixed pulverized material

Physical mixtures (PMs) were prepared by weighing the components at a molar ratio of 1/1 and mixing them using a vortex mixer for 1 min. To prepare 3D ground mixtures (3DGMs), 500 mg of the PM (DPCP/β-CD, DPCP/HPβCD) was weighed and placed in a grinding cell along with 96 g of Φ5 mm alumina balls. Then, the mixtures were processed using a 3D ball mill (3D-80, Nagao system Inc., Kawasaki, Japan) for 30 min.

### Phase solubility diagrams

A solubility phase diagram was constructed following the method described by Higuchi et al. [23]. Excess DPCP (10 mg) was added to aqueous solutions (10 mL) of varying β-CD (0–50 mM) and HPβCD (0–10 mM) concentrations. The mixtures were shaken (25 ± 0.5°C) at 100 rpm for 24 h to obtain a suspension. The suspension was filtered through a 0.20-μm membrane filter (Advantec^®^, Tokyo, Japan), and the filtrate was quantified by high-performance liquid chromatography (HPLC).

The stability constant (*Ks*) was calculated from the slope of the solubility phase diagram and the solubility (*S*_*0*_) of DPCP in the absence of β-CD or HPβCD using the following equation (Eq. 1).

$$K_{S}=\text{slope}/S_{0}\cdot \left(1‐\text{slope}\right)$$ (1)

The complexation efficiency (CE) was calculated using the following equation (Eq. 2).

$$\text{CE}=S_{0}\cdot K_{S}=\text{slope}/\left(1‐\text{slope}\right)$$ (2)

### HPLC conditions

Samples were quantified using HPLC (LC-20ADvp, Shimadzu Corp., Kyoto, Japan). The detection wavelength was set at 293 nm. An Inertsil ODS-3 column (4.6 × 150 mm, Φ5 μm; GL Sciences, Tokyo, Japan) was utilized, with a sample injection volume of 10 μL and a column temperature of 40°C. The mobile phase consisted of acetonitrile and water in a 6:4 ratio. The retention time for DPCP was set at 7 min.

### PXRD measurement

Diffraction intensities were measured using a NaI scintillation counter on a Miniflex II powder X-ray diffractometer (Rigaku Corporation, Tokyo, Japan). Cu-rays (30 kV, 15 mA) were used as the X-ray source. The X-ray diffraction measurements were conducted at a scan speed of 4°/min over a scan range of 2θ = 5–40°. The powder samples were placed flat on a glass plate for measurement.

### FT-IR spectroscopy

Measurements were performed using the attenuated total reflection (ATR) method with a JASCO FT/IR-4600 instrument (JASCO Corporation, Tokyo, Japan). The measurement conditions were as follows: integration frequency, 16; resolution, 4 cm^−1^; and measurement range, 400 cm^−1^–4,000 cm^−1^.

### Scanning electron microscope (SEM) measurements

A field-emission SEM (JSM-IT800, Schottky Field Emission Scanning Electron Microscope, JOEL Ltd., Tokyo, Japan) was used. Each sample was coated with a thin layer of gold for 60 s to enhance conductivity and observed under a pressurized voltage of 1 kV.

### Dissolution test

The dissolution test was conducted using a dissolution tester (NTR-593, Toyama Sangyo, Osaka, Japan) in accordance with the JP18 revised paddle method. The dissolution medium consisted of 300 mL of distilled water maintained at 37 ± 0.5°C, with stirring at 50 rpm. DPCP (50 mg) was placed in a paddle. Samples of 10 mL were collected at 5, 10, 15, 60, and 120 min and, then, filtered through a 0.20-μm membrane filter (DISMIC 25AS, Advantec^®^). To maintain a constant volume of dissolved solution, distilled water (10 mL) was added at the same temperature and volume after each sample collection. The obtained filtrates were diluted with a mixture of acetonitrile and distilled water (6/4), and subsequently analyzed using HPLC.

### ROESY NMR and NOESY NMR measurements

An AVANCE NEO 600 NMR system (Bruker, Billerica, MA, United States) was used. Deuterium oxide (D_2_O) was employed as the solvent, and the measurement conditions were as follows: resonance frequency, 600.13 MHz; measurement temperature, 25°C; integration frequency, 32; pulse width, 90°; and waiting time, 3 s (ROE) and 4 s (NOE).

### Isolation and culture of spleen cells

The spleen was removed from a male C57BL/6 mouse (Tokyo Laboratory Animals Science Co., Ltd., Tokyo, Japan), minced with scissors, and filtered through a 55-μm mesh. Thereafter, the cells were hemolyzed using a red blood cell lysis buffer (Pluriselect-USA Inc., CA, United States) and resuspended in RPMI 1640 medium (Sigma-Aldrich Co., St. Louis, MO, United States) by centrifugation. Isolated splenocytes were confirmed to have >98% viability using trypan blue exclusion assay. Splenocytes (5.0 × 10^6^ cells/mL) were plated and cultured for 48 h in RPMI 1640 medium containing 5% fetal calf serum (GE Healthcare Lifesciences, Inc., IL, United States), 0.01 μg/mL lipopolysaccharide (LPS) (Sigma-Aldrich Co.), and various reagents. The reagents added to splenocytes were CPDP, HPβCD/CPDP, and β-CD/CPDP.

### Measurement of IFN-γ in the culture medium

The IFN-γ concentration in the culture medium (100 μL) was quantified using an ELISA kit (Proteintech Inc., Rosemont, IL, United States), following the manufacturer’s instructions. After treating the culture medium with various reagents for 48 h in primary cultures of splenocytes, the samples were collected and centrifuged at 500 × g for 5 min to remove any particulate matter. Subsequently, the supernatant (100 μL) was used for the quantification of IFN-γ concentration.

### NIH-3T3 cell culture

NIH3T3 (NIH3T3/14-1) mouse fibroblast cells were obtained from Riken BioResource Center (Ibaragi, Japan) and maintained in Dulbecco’s modified eagle medium (DMEM) supplemented with 10% fetal bovine serum and buffered with 44 mM NaHCO_3_ at 37°C in a 5% CO_2_ incubator [24].

For cell injury experiments, a cell suspension was prepared in the aforementioned medium to a concentration of 1.2 × 10³ viable cells/mL. Then, 2 mL of the suspension was seeded onto a 35-mm-φ six-well plate (2.5×10^2^ viable cells/cm^2^). The cells were cultured in a CO_2_ incubator at 37°C with 5% CO_2_ for 96 h. To quantify PGE_2_ concentration in the culture medium, cells were prepared similarly at a concentration of 1.0 × 10^2^ viable cells/mL and, then, seeded in 2-mL portions (2.5 × 10^2^ viable cells/cm^2^) and cultured for 96 h under the same conditions.

### Cell injury experiment

After adding 2 mL of phosphate-buffered saline (PBS) (−) to the NIH-3T3 cells, the dish was irradiated with UV rays from the bottom for 10 s using an 8-m W/cm^2^, 254-nm UV lamp (15 W × 6). Immediately after UV irradiation, PBS (−) with 2.5 mM glucose and 0.9 mM CaCl_2_ was added. The solution was removed by suction and replaced with the conditioning culture medium (PBS (−) with 2.5 mM glucose and 0.9 mM CaCl_2_) to which each drug had been added. The cells were subsequently cultured in a CO_2_ incubator (37°C, 5% CO_2_) for 4 h [25].

### Quantification of PGE_2_ concentration

After adding each drug (to the UV-irradiated NIH-3T3 cells and culturing for 4 h, 50 μL of the conditioning culture medium was sampled from each dish. The concentration of PGE_2_ in the culture medium was measured using an EIA kit (Arbor Assays, Ann Arbor, MI, United States). The PGE_2_ levels were determined colorimetrically at 450 nm. The control groups consisted of cells subjected to UV irradiation without the addition of any drugs, as well as cells without UV irradiation and without drug treatment [26].

### Statistical analysis

The results are presented as means ± standard error of the mean from 3-4 separate experiments for pharmacological measurements. Statistical comparisons between the control and treatment groups were performed using Student's t-test, with significance set at P < 0.05. The statistical analysis was conducted using Statcel-the Useful Addin Forms on Excel (fourth edition; OMS Publishing, Tokyo, Japan).

### Ethics approval

This study was approved by the Institutional Animal Care and Use Committee of the Josai University (No. JU 22037). All procedures involving animals were conducted in accordance with the regulations outlined in the Josai University Guidelines for the Care and Use of Laboratory Animals.

## Results and discussion

### Solubility phase diagram

**FIGURE 2:**
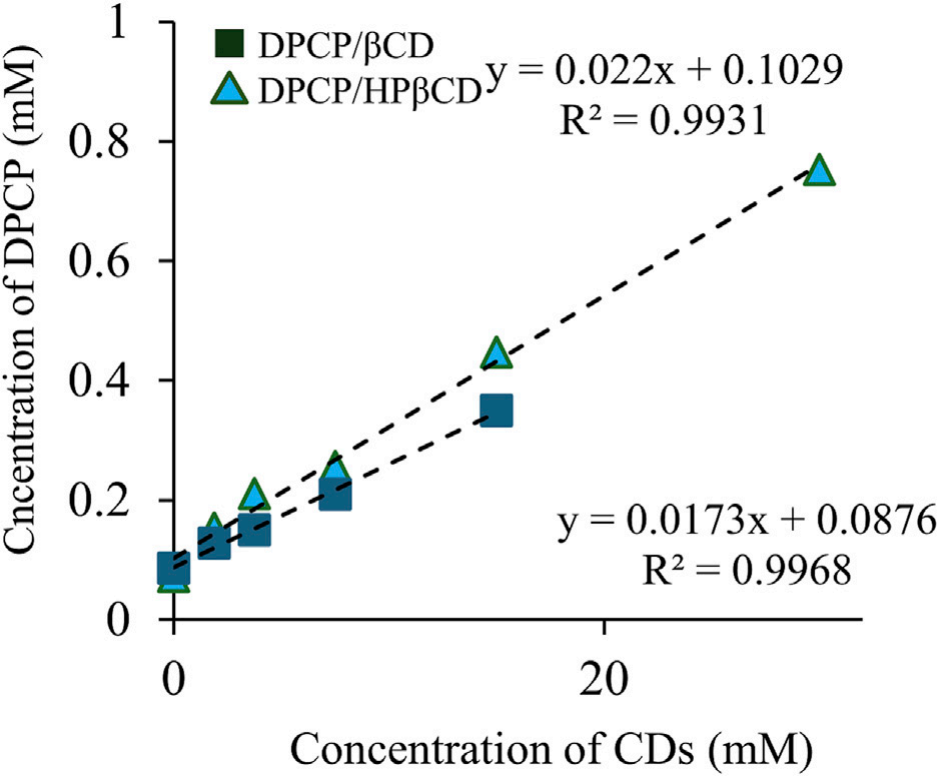
Phase solubility diagram of DPCP/β-CD, DPCP/HPβCD. Results are presented as means±standard deviations (n = 3). ■ DPCP/β-CD, ▲ DPCP/HPβCD DPCP, Diphenylcyclopropenone; HPβCD, hydroxypropyl-β-cyclodextrin; β‐ CD, β-cyclodextrin.

Solubility phase diagrams were constructed to analyze the solubility changes of DPCP/β-CD and DPCP/HPβCD inclusion complexes in solution, which allowed for the calculation of stability constants and prediction of inclusion molar ratios (Figure 2). According to the classification of solubility phase diagrams, they can be categorized into type A (soluble complexes) and type B (insoluble complexes). Type A includes AL (linear diagram), Ap (positive deviation from linearity), and AN (negative deviation from linearity) types, while type B includes BS (the complex has some but limited solubility) and BI (the complex is insoluble) types [27].

DPCP/β-CD, the solubility phase diagram, exhibited a linear AL-type relationship, proportional to the increase in β-CD concentration. The *Ks* was calculated to be 200.4 M^−1^, with a complexation efficiency (CE) of 1.8 × 10^−2^. Similarly, the DPCP/HPβCD solubility phase diagram also showed an AL-type linear relationship, indicating an increase in DPCP solubility proportional to the HPβCD concentration. The *Ks* for DPCP/HPβCD was calculated to be 233.5 M^−1^, with a CE of 2.3 × 10^−2^.

The results from the solubility phase diagrams indicate that both DPCP/β-CD and DPCP/HPβCD form linear AL-type complexes as per the Higuchi et al. classification, suggesting a 1/1 inclusion molar ratio in solution. The higher stability constant for DPCP/HPβCD compared to DPCP/β-CD suggests that DPCP has a greater tendency to form a complex with HPβCD than with β-CD.

### PXRD measurement

**FIGURE 3:**
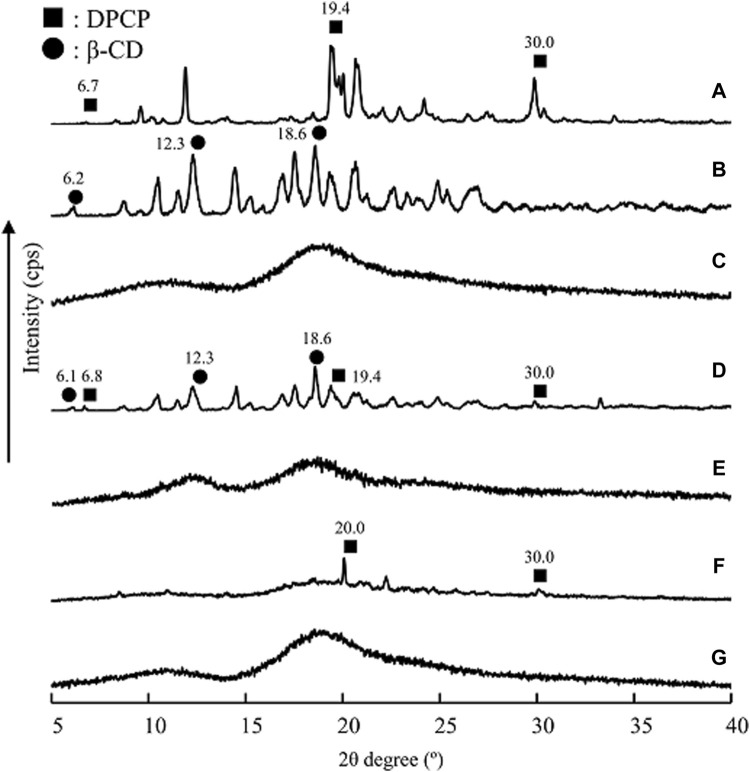
PXRD patterns of DPU intact, DPU/β-CD and DPU/HPβCD systems. **(A)** DPCP intact, **(B)** β-CD intact, **(C)** PM (DPCP/β-CD = 1/1), **(D)** 3DGM (DPCP/β-CD = 1/1), **(E)** HPβCD intact, **(F)** PM (DPCP/HPβCD = 1/1), **(G)** 3DGM (DPCP/HPβCD = 1/1) ■: DPCP original, ▲: β-CD original. 3DGM, three-dimensional ground mixture; DPCP, diphenylcyclopropenone; HPβCD, hydroxypropyl-β-cyclodextrin; PM, physical mixtures; PXRD, Powder X-ray diffraction; β‐ CD, β-cyclodextrin.

The researchers conducted PXRD measurements to examine changes in the crystalline state of solid dispersions of DPCP/β-CD and DPCP/HPβCD that were prepared using 3D milling (Figure 3). Characteristic diffraction peaks for DPCP were observed at 2θ = 6.7°, 19.4°, and 30.0°, while β-CD exhibited characteristic peaks at 2θ = 12.3° and 18.6°. HPβCD displayed a halo pattern without any characteristic diffraction peaks.

For the DPCP/β-CD solid dispersion prepared by physical mixing (PM), both DPCP and β-CD peaks were observed. However, in the 3DGM of DPCP/β-CD, the peaks from DPCP and β-CD disappeared, and a halo pattern was observed. Similarly, in the DPCP/HPβCD solid dispersion PM, the DPCP-derived peak was present, but in the 3DGM of DPCP/HPβCD, both the DPCP- and β-CD-derived peaks disappeared, resulting in a halo pattern.

Previous studies have reported that co-milling can disrupt the crystalline structure, leading to the formation of amorphous solids that exhibit a halo pattern [28]. It has also been suggested that mechanical energy applied during milling and friction can contribute to the formation of amorphous inclusion complexes [29]. Based on the PXRD results, the characteristic diffraction peaks of DPCP, β-CD, and HPβCD were not observed, indicating the formation of inclusion complexes with a presumed molar ratio of 1/1 in the 3DGM of DPCP/β-CD and DPCP/HPβCD.

### FT-IR spectroscopy

**FIGURE 4:**
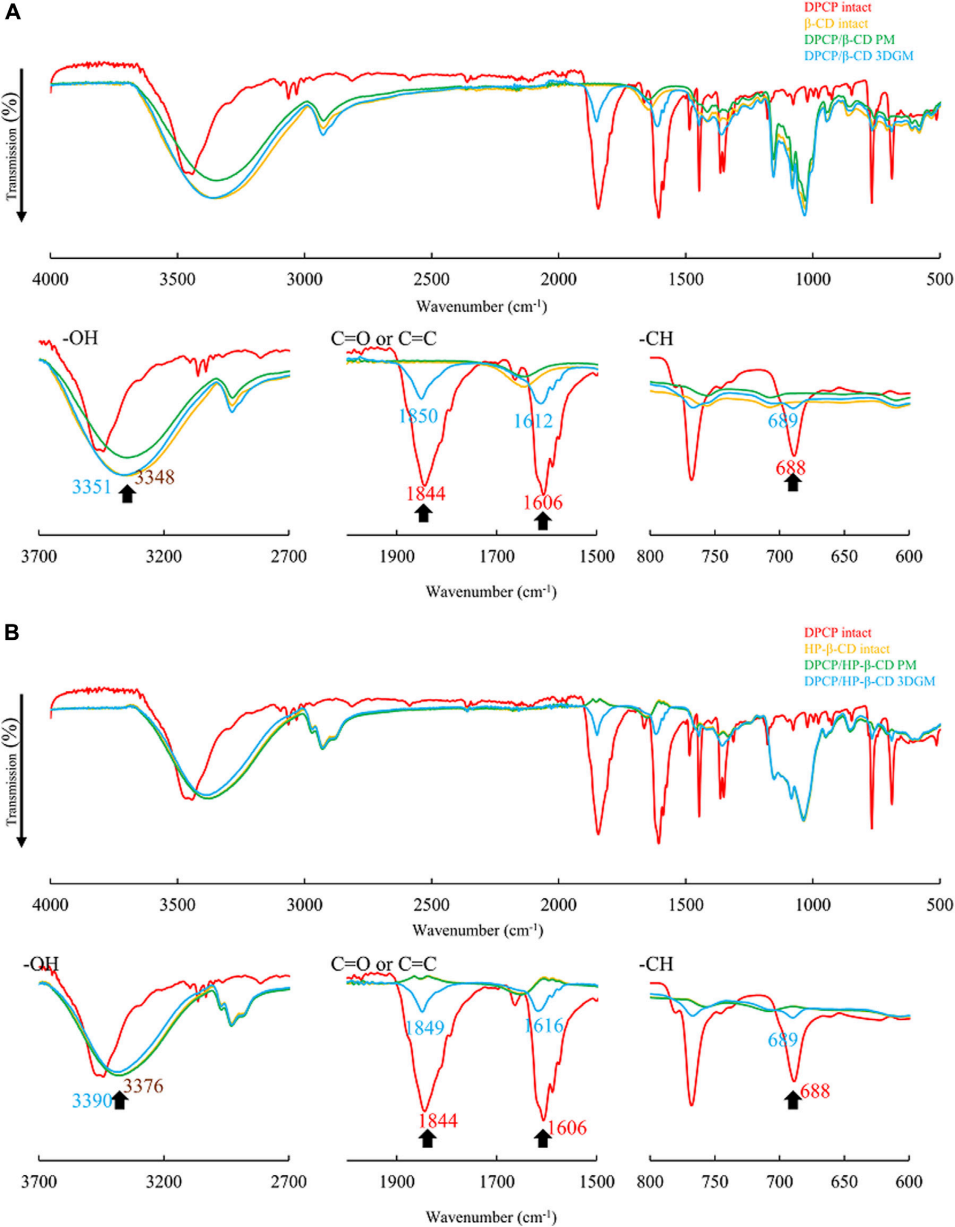
**(A)** FT-IR spectra of DPCP/β-CD systems. FT-IR, Fourier-transform infrared spectroscopy. **(B)** FT-IR spectra of DPCP/HPβCD systems. FT-IR, Fourier-transform infrared spectroscopy; PCP, diphenylcyclopropenone; β‐ CD, β-cyclodextrin.

The PXRD results suggested that DPCP/β-CD and DPCP/HPβCD in the solid state form inclusion complexes with a DPCP/CD molar ratio of 1:1. To further confirm the intermolecular interactions within these complexes, FT-IR absorption spectroscopy was conducted (Figure 4). For DPCP, the FT-IR spectrum exhibited characteristic peaks at 688 cm⁻^1^ for the -CH group derived from the aromatic ring, 1,606 cm⁻^1^ for C=C, and 1844 cm⁻^1^ for the ketone group.

In the physical mixtures (PMs) of DPCP/β-CD and DPCP/HPβCD, the original peaks derived from DPCP and β-CD were observed (Figure 4A). However, in the 3DGM of DPCP/β-CD, shifts were observed in the DPCP-derived peaks: the -CH group peak shifted to 689 cm⁻^1^, the C=C peak to 1,612 cm⁻^1^, and the ketone group peak to 1850 cm⁻^1^. Similarly, in the 3DGM of DPCP/HPβCD, the -CH group peak shifted to 689 cm⁻^1^, the C=C peak to 1,616 cm⁻^1^, and the ketone group peak to 1849 cm⁻^1^ (Figure 4B).

Additionally, in the 3DGM samples, the peaks around 3,348 cm⁻^1^ for β-CD and 3,376 cm⁻^1^ for HPβCD, which correspond to the -OH groups, shifted to 3,351 cm⁻^1^ and 3,390 cm⁻^1^, respectively. The shifts in these peaks, which are attributed to DPCP and β-CD alone, suggest the formation of intermolecular interactions between the C=O and C=C groups of DPCP, the aromatic ring -CH, and the -OH groups of β-CD. These interactions confirm the inclusion complex formation in the solid state through mixed grinding.

### SEM measurements

**FIGURE 5:**
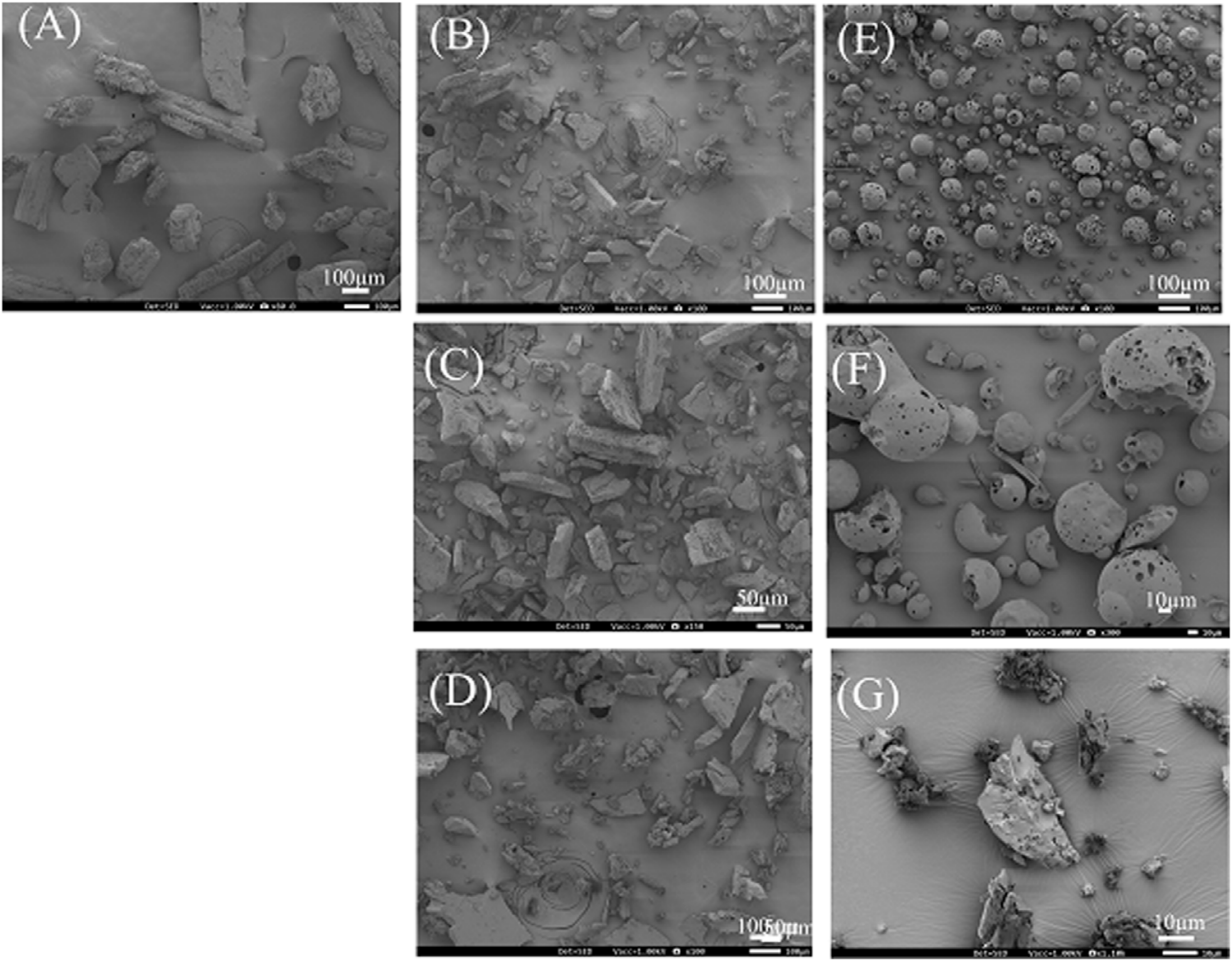
SEM image of DPCP/βCD, DPCP/HPβCD systems. **(A)** DPCP intact, **(B)** β-CD intact, **(C)** PM (DPCP/β-CD = 1/1), **(D)** 3DGM (DPCP/β-CD = 1/1), **(E)** HPβCD intact, **(F)** PM (DPCP/HPβCD = 1/1), **(G)** 3DGM (DPCP/HPβCD = 1/1) 3DGM, three-dimensional ground mixture; DPCP, diphenylcyclopropenone; HPβCD, hydroxypropyl-β-cyclodextrin; PM3DGM, three-dimensional ground mixture, physical mixtures; PXRD, Powder X-ray diffraction; β -CD, β-cyclodextrin.

In the solid state, 3DGMs of DPCP/β-CD and DPCP/HPβCD, both with a 1:1 M ratio, were suggested to form inclusion complexes. PXRD measurements indicated changes in the crystal state of these complexes. To evaluate the microstructure and surface morphology of the prepared inclusion complexes, SEM measurements were performed. SEM, with its high resolution, can observe fine structures on the nanometer scale, providing detailed insights into the microstructure and surface characteristics of the materials (Figure 5).

The SEM images revealed that the crystal surface of intact DPCP appeared smooth and columnar, with particle sizes around 100 μm (Figure 5A). Intact β-CD displayed a smooth and angular crystal surface with particle sizes around 150 μm (Figure 5B). In the PM of DPCP/β-CD (1/1), no significant changes in the particle surfaces of DPCP and β-CD were observed (Figure 5C).

Generally, when β-CD forms inclusion complexes, cubic crystals are reported to appear. In the 3DGM of DPCP/β-CD (1/1), the particle size was reduced to around 100 μm due to milling, and the particles exhibited a cubic shape with fine surface attachments, suggesting the formation of an inclusion complex (Figure 5D).

HPβCD intact exhibited a smooth and spherical crystal surface with particle sizes around 100 μm (Figure 5E). In the PM of DPCP/HPβCD (1/1), the particle surfaces of DPCP and HPβCD remained unchanged (Figure 5F). Typically, when HPβCD forms inclusion complexes, crystals with a folded structure are observed [30]. In the 3DGM of DPCP/HPβCD (1/1), the particle size was further reduced to approximately 30 μm, and a plate-like folded structure with fine particles on the surface was observed, indicating the formation of an inclusion complex (Figure 5G).

These SEM observations suggest that the particle shapes and surface morphologies changed due to the formation of inclusion complexes through the 3DGM process, confirming the successful inclusion of DPCP with β-CD and HPβCD.

### Dissolution test

**FIGURE 6:**
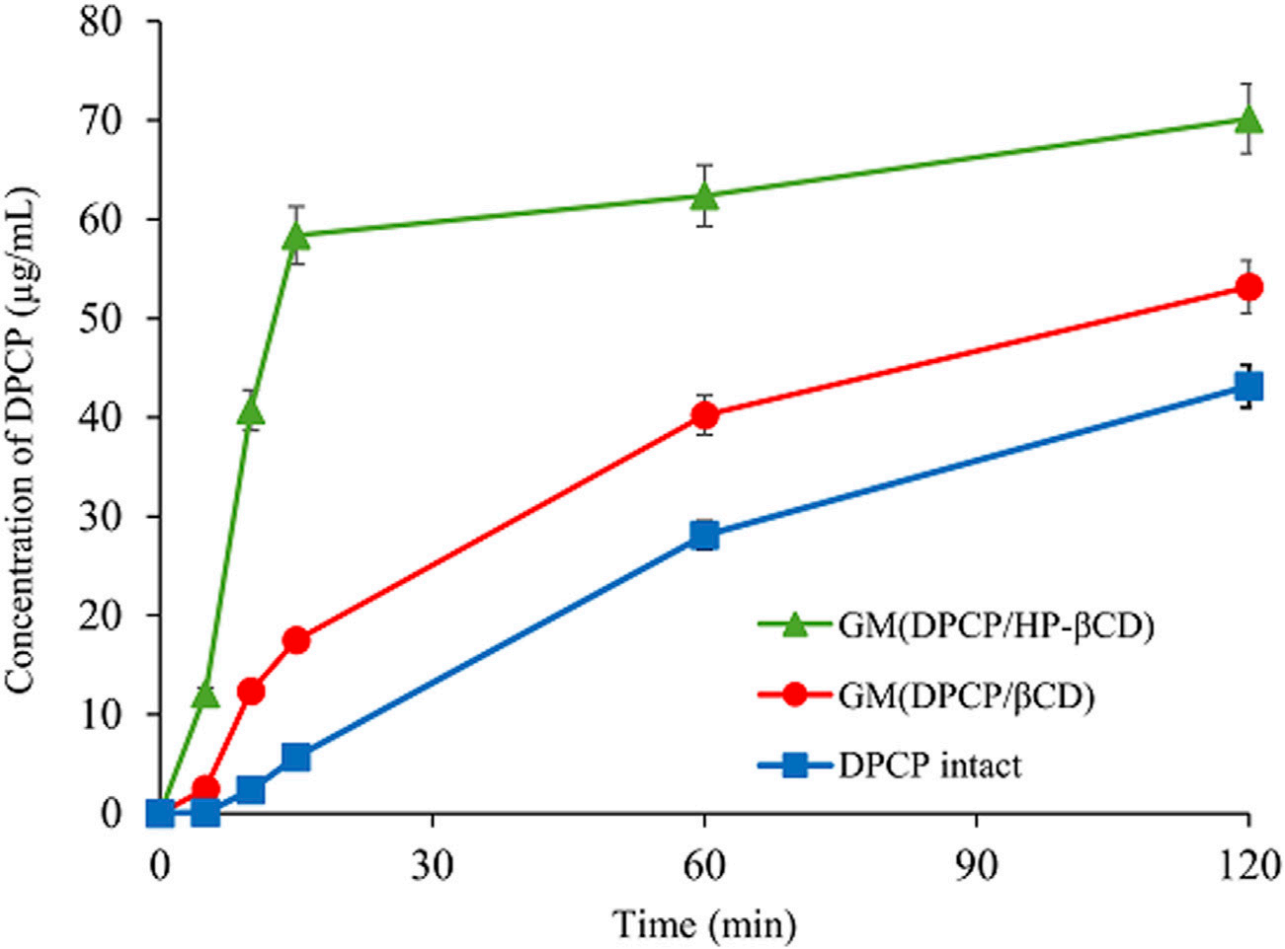
Dissolution profiles of DPCP intact, DPCP/β-CD and DPCP/HPβCD systems. Results are presented means±standard deviations (n = 3). DPCP, diphenylcyclopropenone; HPβCD, hydroxypropyl-β-cyclodextrin; β‐ CD, β-cyclodextrin.

Solid-state characterization confirmed the formation of inclusion complexes in 3DGMs of DPCP/β-CD (1/1) and DPCP/HPβCD (1/1). To further investigate the change in solubility of DPCP with each inclusion complex, dissolution tests were conducted using samples of intact DPCP, 3DGM (DPCP/β-CD = 1/1), and 3DGM (DPCP/HPβCD = 1/1) (Figure 6).

Initially, the dissolution concentration of intact DPCP at 15 min was measured at 5.70 μg/mL. In contrast, both 3DGM (DPCP/β-CD = 1/1) and 3DGM (DPCP/HPβCD = 1/1) exhibited faster dissolution of DPCP compared to DPCP alone, with concentrations measured at 17.5 μg/mL and 58.4 μg/mL, respectively, from study initiation. This significant increase in the solubility of DPCP in the presence of inclusion complexes can be attributed to two main factors: the formation of inclusion complexes between DPCP and β-CD or HPβCD, and the increase in specific surface area due to the smaller particle size achieved through milling.

Notably, the solubility of DPCP was higher in 3DGM (DPCP/HPβCD = 1/1) compared to that in 3DGM (DPCP/β-CD = 1/1). This suggests that there may be differences in the inclusion mode or interaction strength between DPCP/β-CD and DPCP/HPβCD complexes, in addition to the findings from the physical properties evaluation in the solid state.

### ROESY NMR and NOESY NMR measurements

**FIGURE 7:**
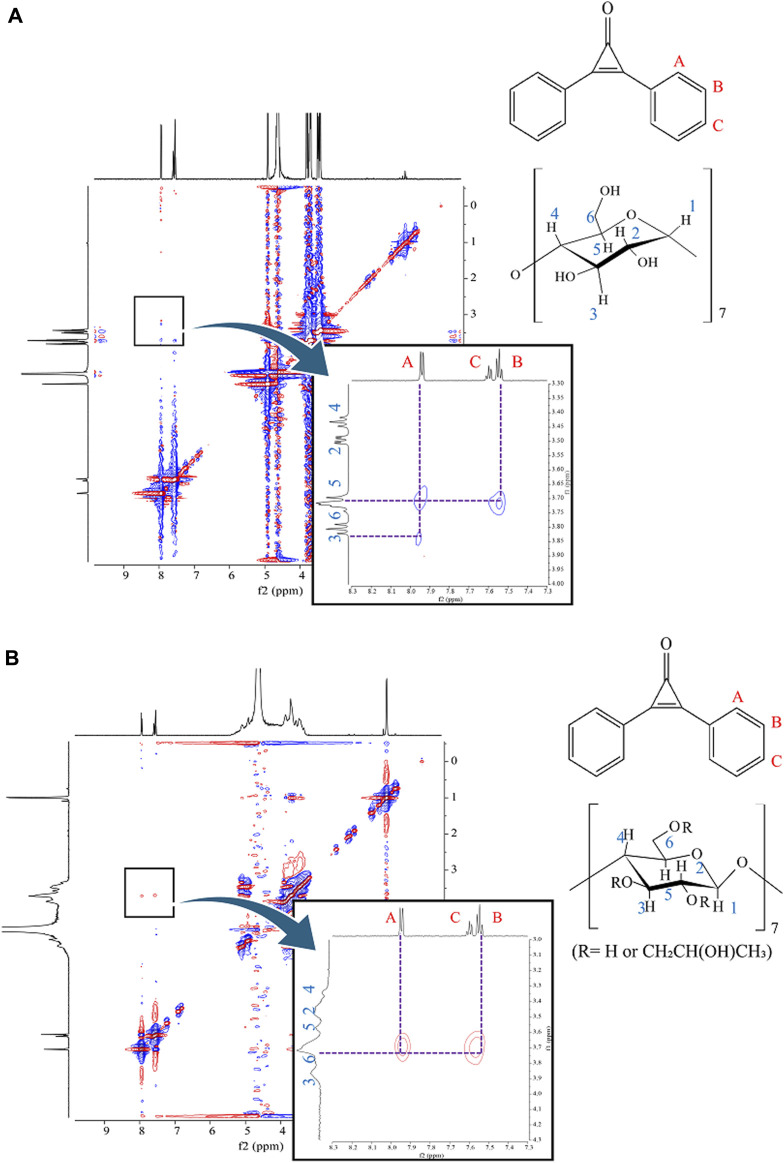
**(A)** ^1^H-^1^H ROESY NMR spectra of 3DGM (DPCP/β-CD = 1/1) systems. 3DGM, three-dimensional ground mixture; DPCP, diphenylcyclopropenone; NMR,nuclear magnetic resonance; β -CD, β-cyclodextrin. **(B)** ^1^H-^1^H NOESY NMR spectra of 3DGM (DPCP/HPβCD = 1/1) systems.

NMR spectroscopy was employed to elucidate the inclusion modes of DPCP with β-CD and HPβCD in the prepared 3DGM at a 1:1 mole ratio. In the case of 3DGM (DPCP/β-CD = 1/1), cross-peaks were identified between the -CH (A; 7.94 ppm) of DPCP and the -OH groups of β-CD (H-6; 3.71 ppm, H-3; 3.82 ppm), as well as between -CH (B; 7.55 ppm) of DPCP and -OH (H-6; 3.71 ppm) of β-CD (Figure 7A) [31–33]. These findings indicate that DPCP is encapsulated by β-CD from the wide edge (H-3) to the narrow edge (H-6), encompassing the benzene ring of DPCP.

Conversely, in 3DGM (DPCP/HPβCD = 1/1), cross-peaks were observed between -CH of DPCP (A; 7.95 ppm) and -OH of HPβCD (H-6; 3.72 ppm), as well as between -CH (B; 7.55 ppm) of DPCP and -OH (H-6; 3.72 ppm) of HPβCD (Figure 7B). These results from ROESY and NOESY NMR measurements suggest that HPβCD encapsulates DPCP such that the CD surrounds the benzene ring of DPCP from the narrow edge (H-6) to the wide edge (H-3).

The distinct cross-peak patterns observed in the NMR spectra of DPCP/β-CD and DPCP/HPβCD complexes indicate different inclusion modes between DPCP and the CDs in solution. Specifically, DPCP/β-CD predominantly interacts with the -OH groups of β-CD, while DPCP/HPβCD interacts in a manner where HPβCD surrounds the benzene ring of DPCP.

These findings further support the hypothesis that the 3DGM preparation method affects the inclusion mode and interaction strength of DPCP with β-CD and HPβCD, influencing their solubility enhancement properties observed in dissolution tests.

### Measurement of IFN-γ in the culture medium

**FIGURE 8:**
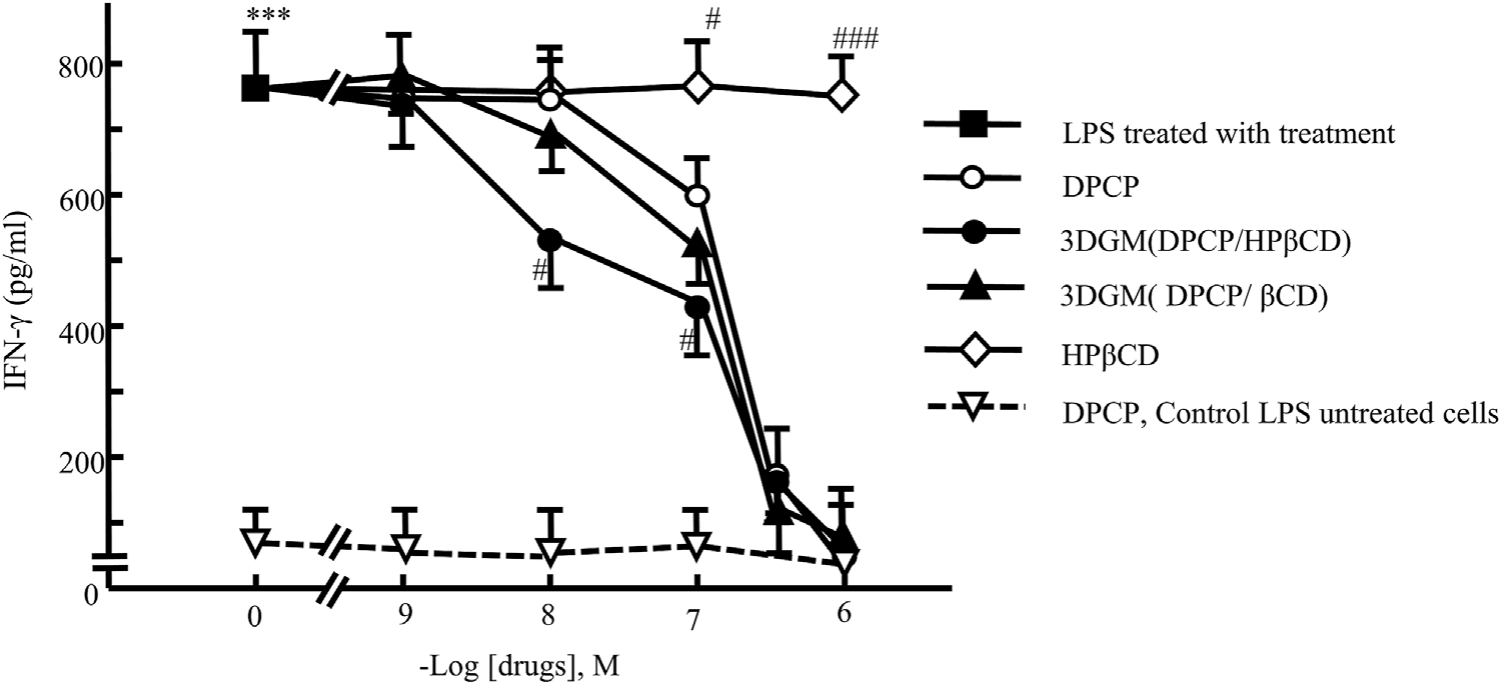
Dose-response relationship of DPCP and its inclusion compounds on LPS-induced IFN-γ secretion in primary cultured splenocytes: IFN-γ concentration (pg/mL) Cell seeding density: 5.0 × 10^6^ cells/mL, data: means±standard errors of the mean (n = 3-4) ***(P < 0.001) indicate significant differences from the control (medium alone) group, and #(P < 0.05), ##(P < 0.01) and ###(P < 0.01)indicate significant differences from the LPS + DPCP group. DPCP, diphenylcyclopropenone; HPβCD, hydroxypropyl-β-cyclodextrin; LPS, lipopolysaccharide; NMR, nuclear magnetic resonance; β‐ CD, β-cyclodextrin.

In this study, 3DGM samples indicated that DPCP/β-CD and DPCP/HPβCD form inclusion complexes at a molar ratio of 1/1, albeit with distinct inclusion modes. DPCP is recognized for its ability to reduce elevated serum IFN-γ levels in patients with AA [34]. Thus, we investigated the anti-inflammatory effects of DPCP inclusion complexes (DPCP/β-CD and DPCP/HPβCD) using LPS-induced IFN-γ as a model to assess their efficacy (Figure 8).

The results demonstrated that DPCP alone significantly decreased LPS-induced IFN-γ levels at a concentration of 10^−7^ M compared to the control group. Conversely, β-CD and HPβCD alone did not show any effect on LPS-induced IFN-γ levels. Furthermore, both DPCP/β-CD and DPCP/HPβCD inclusion complexes significantly attenuated LPS-induced IFN-γ at a DPCP concentration of 10^−7^ M compared to the control group. Particularly, HPβCD/DPCP exhibited a more pronounced reduction in LPS-induced IFN-γ at lower concentrations (10^−8^ M), indicating a stronger inhibitory effect than that observed with DPCP alone or DPCP/β-CD. Additionally, the HPβCD group without encapsulated DPCP did not influence the LPS-induced IFN-γ levels.

These findings underscore that DPCP/β-CD and DPCP/HPβCD exert differential effects on reducing LPS-induced IFN-γ levels. The enhanced efficacy of HPβCD/DPCP suggests potential advantages in therapeutic applications aimed at mitigating conditions associated with elevated IFN-γ, such as AA.

### Cell injury experiment

**FIGURE 9:**
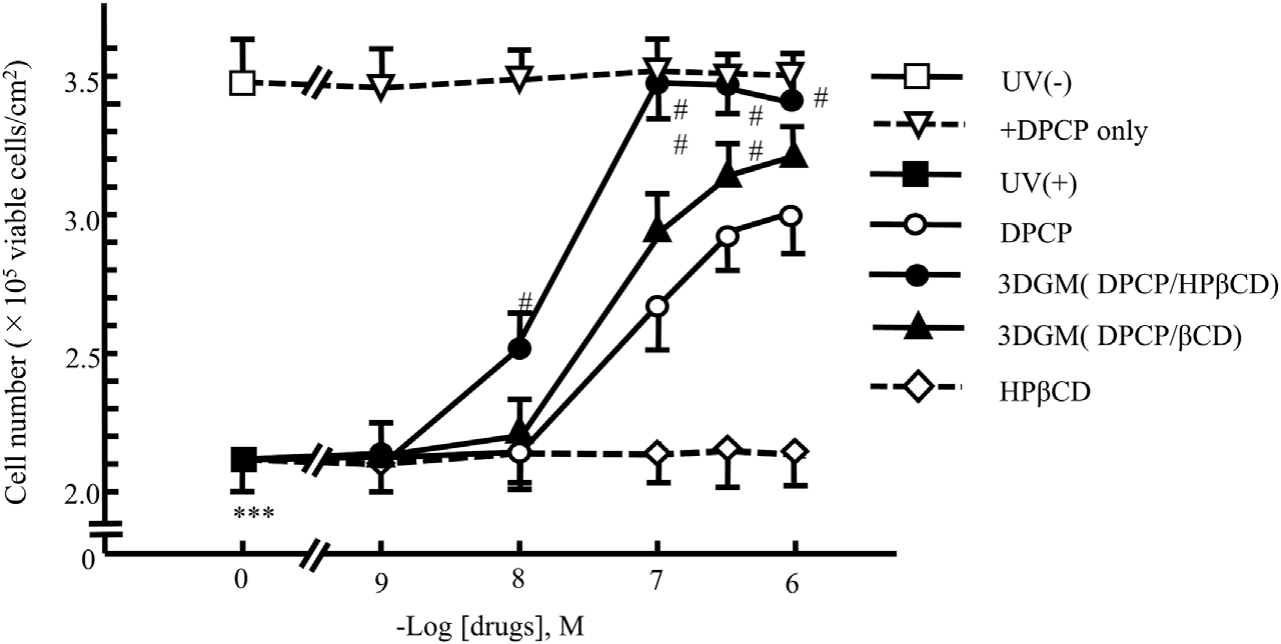
Dose-response relationship of DPCP and its inclusion compounds on UV-radiation-induced cell damage in NIH-3T3 cells cultured for 4 h Cell seeding density: 2.5 × 10^2^ cells/cm^2^, data: means ± standard errors of the mean (n = 3-4) ***(P < 0.001) indicate significant differences from the control [medium alone: UV (−)] group, and #(P < 0.05) and ##(P < 0.01) indicate significant differences from each UV (+)+DPCP group. DPCP, diphenylcyclopropenone; UV, ultraviolet.

We investigated the protective effects of each drug on UV-irradiated NIH-3T3 cells subjected to UV damage (Figure 9). Following 4 h of UV irradiation, the number of viable NIH-3T3 cells decreased to approximately 70% compared to that of the untreated group (no UV irradiation, no drug added). In each drug-treated group, the decrease in cell viability was significantly attenuated in a dose-dependent manner.

The group supplemented with DPCP showed a dose-dependent suppression of UV-induced cell damage, with recovery to 85% of the non-UV-irradiated group observed at 10^−6^ M DPCP. Similarly, the β-CD/DPCP group, where DPCP was encapsulated in β-CD, exhibited a comparable protective effect against cell damage. In contrast, the HPβCD/DPCP group showed significant inhibition of cell damage starting from 10^−8^ M HPβCD/DPCP, with recovery to 97% of the non-UV-irradiated group at 10^−7^ M HPβCD/DPCP.

**FIGURE 10:**
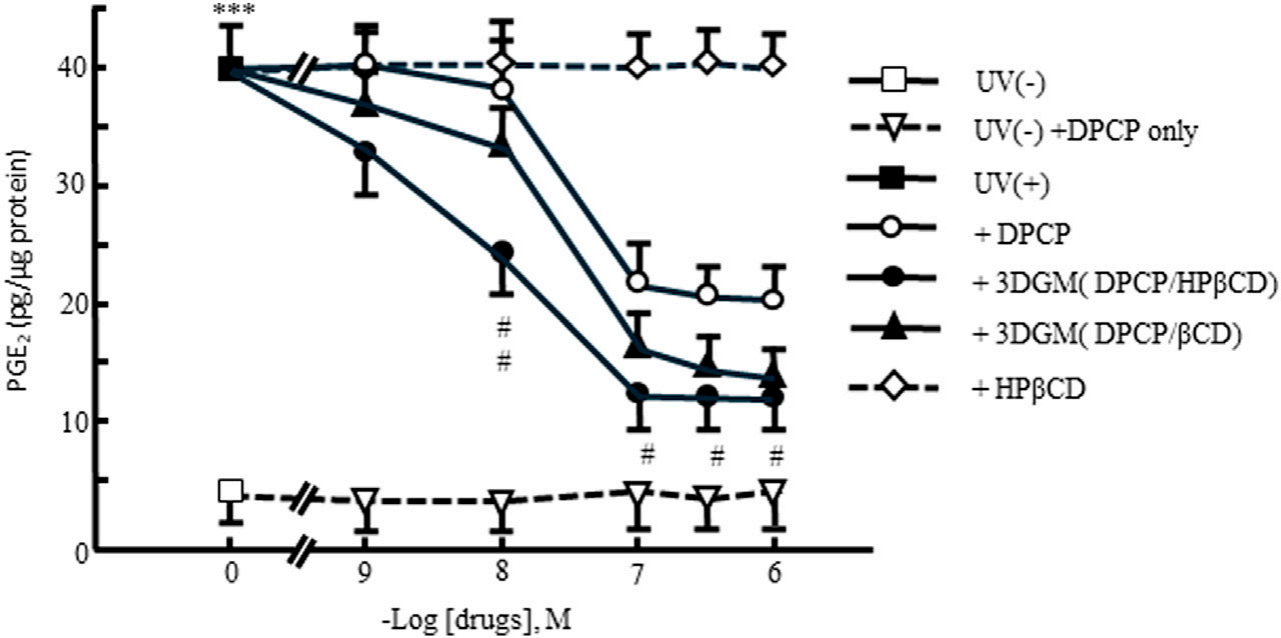
Dose-response relationship of DPCP and its inclusion compounds on UV-radiation-induced prostaglandin E_2_ release in NIH-3T3 cells cultured for 4 h Cell seeding density: 2.5 × 10^2^ cells/cm^2^, data: means±standard errors of the mean (n = 3-4) ***(P < 0.001) indicate significant differences from the control [medium alone: UV(−)] group, and #(P < 0.05) and ##(P < 0.01) indicate significant differences from each UV(+)+DPCP group. DPCP, diphenylcyclopropenone; UV, ultraviolet.

Furthermore, the release of PGE_2_ into the culture medium of UV-irradiated NIH-3T3 cells after 4 h was evaluated (Figure 10). PGE_2_ is a known chemical mediator involved in inflammation following UV radiation exposure. The addition of DPCP and its inclusion complexes led to a significant increase in PGE_2_ release compared to the untreated group. However, DPCP and its complexes also showed a dose-dependent suppression of PGE_2_ release induced by UV radiation. At 10^−-6^ M DPCP, PGE_2_ release was reduced to 50% of the UV-irradiated control, while β-CD inclusion and HPβCD groups exhibited inhibitory effects at lower concentrations. Notably, the HPβCD group at 10^−7^ M suppressed PGE_2_ release to 25% of the UV-irradiated control, indicating a potent anti-inflammatory effect.

These results suggest that the addition of DPCP and its complexes protects UV-damaged NIH-3T3 cells, preserves cell viability, and reduces PGE_2_ release, which is indicative of anti-inflammatory activity. The enhanced efficacy observed with HPβCD/DPCP compared to β-CD/DPCP may be attributed to differences in the inclusion modes of these complexes, highlighting the importance of inclusion complex formation in modulating biological activity.

The *ex vivo* cell studies aimed to validate whether inclusion complexes with β-CD derivatives could preserve the anti-inflammatory effects of DPCP. Results from these studies confirmed that both β-CD and HPβCD inclusion complexes maintained the anti-inflammatory properties of DPCP. Particularly noteworthy was the finding that the DPCP/HPβCD complex exhibited anti-inflammatory effects at lower concentrations compared to the DPCP/β-CD complex, which represents a novel discovery. This difference in inclusion modes, elucidated through NOESY NMR measurements, suggests a potential pharmacological advantage where DPCP demonstrates enhanced anti-inflammatory efficacy.

However, the safety and pharmacokinetic profiles of DPCP/β-CD inclusion complexes, which typically require comprehensive *in vivo* studies, remain a limitation of this research and should be addressed in future investigations.

The study unequivocally demonstrated that DPCP/β-CD complexes enhance solubility and retain anti-inflammatory properties. A clinical challenge highlighted is the current use of acetone, an organic solvent, in preparing DPCP for AA treatment. Based on these findings, future efforts will focus on scaling up the preparation method using 3D ball milling and exploring alternative formulation methods. Addressing the stability of DPCP/β-CD complexes in both solid-state and solution phases remains a critical challenge that requires further investigation.

As this study focused primarily on improving the solubility of DPCP, future research directions should include investigating skin permeability for potential clinical applications, thereby addressing the broader implications of these findings.

## Conclusion

In this study, DPCP/β-CD and DPCP/HPβCD inclusion complexes were successfully prepared using the 3DGM method, which eliminates the need for organic solvents. Dissolution tests confirmed enhanced solubility of DPCP in both 3DGM (DPCP/β-CD = 1/1) and 3DGM (DPCP/HPβCD = 1/1) complexes. NMR analysis indicated that these complexes formed at a 1:1 molar ratio but exhibited distinct inclusion modes between DPCP/β-CD and DPCP/HPβCD. Notably, 3DGM (DPCP/HPβCD) demonstrated superior anti-inflammatory activity at lower doses compared to 3DGM (DPCP/β-CD), suggesting enhanced effectiveness of DPCP when complexed.

These findings hold promise for optimizing DPCP’s handling characteristics and mitigating potential side effects. Improving DPCP’s solubility through these complexes could expand its applications in various fields, including dermatology for treating conditions, such as AA. Moreover, this research underscores the importance of considering skin irritation potential in formulation development, crucial for ensuring patient safety and comfort.

By advancing the formulation of DPCP through innovative methods, such as 3D ball milling, this study enhances therapeutic efficacy and sets the stage for future clinical applications and formulation improvements.

## Data Availability

The original contributions presented in the study are included in the article/supplementary material, further inquiries can be directed to the corresponding author.

## Abbreviations

3DGM, 3D ground mixture; AA, Alopecia areata; ATR, attenuated total reflection; CE, Complexation Efficiency; DPCP, diphenylcyclopropenone; FT-IR, Fourier-transform infrared spectroscopy; HPβCD, hydroxypropyl-β-cyclodextrin; IFN-γ, interferon gamma; LPS, lipopolysaccharide; NMR, ^1^H nuclear magnetic resonance; PBS, phosphate-buffered saline; PGE, prostaglandin E; PM, physical mixture; PM, physical mixture; PXRD, powder X-ray diffraction; SEM, scanning electron microscope; β-CD, β-cyclodextrin.
